# Do You Have Problems When Reproducing Bioactivities of Food or Food Components? The Importance of Biological Rhythms

**DOI:** 10.3390/nu14214607

**Published:** 2022-11-02

**Authors:** Anna Arola-Arnal, Manuel Suárez

**Affiliations:** Nutrigenomics Research Group, Departament de Bioquímica i Biotecnologia, Universitat Rovira i Virgili, 43007 Tarragona, Spain

With the onset of omics sciences, in the 20th century, nutritional studies evolved to investigate the effects of diet at a molecular level, giving rise to nutritional genomics, which includes both nutrigenomics and nutrigenetics [1]. This fact changed the focus of the field of nutrition, generating an incredible amount of data regarding the interaction of nutrients and food components with genes. The development of new tools to analyze, quantify, and process the data significantly contributed to advancements in this field of research. Simultaneously, evidence about the importance of eating timing and dietary patterns grew, thus highlighting that circadian rhythms modulate many aspects of physiology. In this regard, the knowledge of the molecular basis of these rhythms has rapidly expanded in recent years, as well as knowledge of the interaction of nutrition on their regulation. Altogether, a new field of nutrition, known as chrononutrition, has emerged [2].

This new research field focuses on the relationship between biological rhythms, nutrition, and metabolism. It has been shown that these factors have a direct impact on human health. Indeed, the disruption of biological rhythms is believed to be an element of the onset of metabolic diseases such as metabolic syndrome [3]. On the other hand, it has been reported that some components of diet, such as phenolic compounds, can interact with the molecular clocks, thus contributing to the maintenance of the rhythms [4]. In this regard, some studies have already shown that phenolic compounds from grape seed extract (GSPE), only when administered at night, enhanced the energy profile and improved mitochondrial function and oxidation in the liver in diet-obese male Fischer 344 rats [5]. Interestingly, in these animals, the bioavailability of the phenolic compounds of this extract was dependent on the moment of administration, increasing the amount of some microbial metabolites when administered at night [6]. Unfortunately, there are not many studies that focus on the evaluation of the impact on the consumption of bioactive compounds under different photoperiods. In this regard, some studies have evaluated the effect of the consumption of phenolic-rich fruits including cherries, oranges, tomatoes, and grapes. The results have shown that bioavailability and metabolic effects are dependent on the photoperiod of consumption [7,8,9,10,11,12].

In this sense, the collection of manuscripts presented in this Special Issue sheds some light on different aspects of biological rhythms and nutrition in relation to the maintenance of health. Arreaza-Gil et al. [13] show that the photoperiod modulates the composition of gut microbiota, especially in obesity, and these changes can be related to several physiological parameters such as body weight gain and adipose tissue distribution. Indeed, regarding adipose tissue, Ribas-Latre and Eckel-Mahan [14] review the importance of the peripheral clocks located in adipose tissue in the maintenance of health. Focusing on nutrition, the consumption of fruits (cherries and tomatoes) has been shown to exert a photoperiod-dependent effect. For instance, in cherries, some plasma metabolic parameters, such as triacylglycerides and the hepatic expression of genes from the lipid metabolism, were changed across the photoperiod [15]. Comparably, in tomatoes, the seasonal daylight schedule affected the bioavailability of their phenolic compounds, which could partially explain the different results obtained among the photoperiods [16]. These results highlight the importance of seasonality and the photoperiod of consumption on the metabolic effects of fruits. Finally, phenolic extracts such as GSPE mitigate the disturbances caused by an abrupt photoperiod change in rats, thus contributing to the adaptation to new photoperiods [17].

All these results, as well as those previously published by other authors, raise the question of whether the impact on the health of food or food components could be different if the experiments had been carried out at different administration times, including both circadian and circannual points. Indeed, many of the articles usually do not report the moment of administration of the bioactives under study or even the light/dark cycles during housing. For instance, some studies do not control administration time and add the bioactive compounds into the feed, thus losing the capacity to determine the actual moment of administration. All these factors could underlie the wide array of results and effects observed among the effects of different bioactive compounds in animal studies. The problem is worst when moving to clinical studies, where the timing of consumption of bioactive compounds is not easily controlled, introducing an important confounding factor into the results, thus contributing to increasing variability, hindering the identification of effects among different experimental groups, or even the reproducibility of the studies.

Altogether, the evidence shows that biological rhythms play a crucial role in the regulation of the metabolism. Therefore, guidelines considering the moment of administration and the light/dark cycles when planning and reporting animal and human studies are essential to be able to obtain clear conclusions from the studies. Ultimately, dietary recommendations, as well as those concerning nutraceuticals and functional foods, must advise consumers about the moment of intake to maximize the derived health effects.
